# A New Questionnaire for Estimating the Severity of Visual Height Intolerance and Acrophobia by a Metric Interval Scale

**DOI:** 10.3389/fneur.2017.00211

**Published:** 2017-06-01

**Authors:** Doreen Huppert, Eva Grill, Thomas Brandt

**Affiliations:** ^1^Institute for Clinical Neurosciences, German Center for Vertigo and Balance Disorders, Ludwig-Maximilians-University Munich, Munich, Germany; ^2^Institute for Medical Information Processing, Biometrics and Epidemiology, Ludwig-Maximilians-University Munich, Munich, Germany

**Keywords:** fear of heights, visual height intolerance, acrophobia, questionnaire, metric interval scale, Rasch analysis

## Abstract

**Aims:**

To construct and validate a short scale for the assessment of the severity of visual height intolerance (vHI) and acrophobia.

**Methods:**

The questionnaire was developed from two earlier representative epidemiological studies (*n* = 5,529). Items were applied in a telephone survey of a representative population-based sample.

**Results:**

A total of 1,960 persons were included. The life-time prevalence of vHI was 32.7% (f: 36.1%; m: 28.4%); 12% of these persons fulfilled the psychiatric criteria of acrophobia. Rasch analysis of 11 items on severity, symptoms, and triggers resulted in an 8-item scale with good fit to the model. The score differentiated well between persons with and without acrophobia. The distribution of the scores on the metric scale of the questionnaires of those individuals with acrophobia is separate and distinct from that of susceptibles without acrophobia, although there is some overlap.

**Conclusion:**

Our proposed short questionnaire (vHISS, see Table 1 and Supplementary Material) allows a continuous quantification of the severity of vHI within a metric interval scale from 0 to 13. The diagnosis of acrophobia can be established by including two additional questions.

## Introduction

Epidemiological studies usually focus on a definition of fear of heights as a subtype of specific phobias. The criteria of fear of heights are based on the diagnostic features of anxiety and panic attacks elicited by exposure to heights (1). The frequency of fear of heights is reported to have a life-time prevalence of 3.1–6.4% (2–4). A cross-sectional epidemiological study on 3,517 individuals representative of the general population revealed that the life-time prevalence of visual height intolerance (vHI) of varying degrees of severity, including fear of heights, amounts to 28% (women 32%, men 25%) (5). A subsequent study (*n* = 2,012) on clinical characteristics almost exactly replicated the life-time prevalence of vHI (28.5%; women 32.4%, men 24.5%) (4). There is an inter- and intraindividual continuum stretching from fear of heights (acrophobia) to a less-pronounced vHI, to which the category of a specific phobia does not apply. Physiological visual height imbalance (prevalence 100%) should be distinguished from vHI (prevalence 28%; clinical relevance in about 50%) and acrophobia (prevalence about 5%), a specific phobia (1). The latter diagnosis is defined in the ICD-10 (6) and DSM-V (7) by the following criteria: an intense fear; avoidance of exposure to heights; one of the vegetative symptoms like trembling, palpitation, sweating, inner agitation; and two other symptoms from the diagnostic symptom list. A time criterion of at least 6-month duration is required. The non-medical Anglo-American community uses the same term to refer to a more or less-pronounced vHI that does not generally fulfill the above criteria.

Questionnaires used up to now to validate susceptibility to fear of heights either compare self-reports and overt-behavioral procedures (8) or measure height-relevant interpretation biases to help assess the relationship between biased interpretations and symptoms of acrophobia (9). A reliable questionnaire is not available for evaluating susceptibility to and severity of vHI, including acrophobia. It was our aim to construct and validate an easy-to-handle short questionnaire that can be used for a reliable evaluation of susceptible individuals.

When developing a scale, the specific objectivity of its candidate items should be taken into account. One of the most relevant properties of an outcome measure is its specific objectivity. This property assumes that an easy item is more likely to be completed than a difficult item, and that a person with high ability is more likely to complete an item than a person with low ability. Specific objectivity also means that the single sum score is able to measure the underlying trait of the person and that this score is valid irrespective of the abilities of the observed population and the difficulty of the items. This ensures that all the items included in the scale measure the same trait. Also, by testing specific objectivity, redundant items can be identified, i.e., items that do not contribute to knowledge about the person’s abilities and can be easily dropped, thus leading to a more concise scale. The benefit of such a quantitative questionnaire of susceptibility to heights is that it allows at least five applications:

- provides inclusion criteria and characteristics for susceptible individuals for scientific investigations and clinical trials;
- estimates the severity and the medical necessity of a recommended therapy;
- assesses quantitatively the spontaneous course or the effectiveness of therapy in follow-up studies;
- weighs the implications for potential professional restrictions;
- detects psychiatric comorbidity in susceptibles.

The Objective of this study was to validate a questionnaire that yields a quantitative measure of the severity of vHI. In the following, we present a short and robust questionnaire (Table 1; Supplementary Material: the downloadable English and German versions of Table 1) that is both easy to perform and to interpret. This questionnaire provides a metric interval scale rather than a mere grouping by different grades of severity.

**Table 1:** **A metric interval scale for estimating the severity of visual height intolerance (vHI)**.

Visual Height Intolerance Severity Scale (vHISS)		
**Question**		
Have you already experienced vHI while looking from a height? (distressing instability when standing or moving)	Yes □ 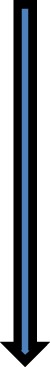	No □ 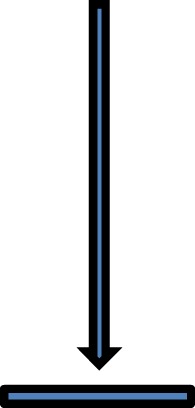
Continue to fill out the rest of the questionnaire only if you answered “yes.”	Continue	Finished

1. Because of your vHI, how much difficulty did you recently have doing sports?	0 □	No difficulty
	1 □	Any difficulty (a little/moderately/quite a lot/very much)

2. Because of your vHI, how much difficulty did you recently have in your daily activities?	0 □	No difficulty
	1 □	A little
	2 □	Moderately/quite a lot/very much

3. Because of your vHI, how much is your quality of life affected?	0 □	Not at all
	1 □	A little
	2 □	Moderately/quite a lot/very much

4. I have vHI when exposed to heights	0 □	… occasionally
	1 □	… often/frequently/always

5. Now I have vHI that is …	0 □	… less strong than before
	1 □	… just as strong as before
	2 □	… stronger than before

6. I have/had vHI for longer than 6 months	0 □	No
	1 □	Yes
**List A**		
7. What *bodily* symptoms do you feel when exposed to heights? (multiple answers possible)	□	a. Trembling
	□	b. Palpitations
	□	c. Inner agitation
	□	d. Sweating/moist hands
	□	e. Light-headedness
	□	f. Postural (to-and-fro) dizziness
	□	g. Weakness in the knees
	□	h. Instability of stance and gait
	□	i. Malaise/queasy feeling in the stomach
	□	j. Oppression
	□	k. Fearfulness
	□	l. Mental image of falling
	□	m. Gait disorder
	□	n. Others ………
	□	None of the above

**List B**		
8. vHI is induced by my … (multiple answers possible)	□	Standing on or climbing up a tower
	□	Standing on or walking over a bridge
	□	Standing on or walking up steps
	□	Standing on or climbing up a ladder
	□	Standing on or walking on a balcony
	□	Looking out of a window
	□	Standing or walking on a scaffolding
	□	Standing or walking on a roof
	□	Riding on a carousel or a Ferris wheel
	□	Riding in a ski lift or gondola
	□	Hiking/mountain climbing
	□	Rock climbing
	□	Other situations. If yes, please name
	□	…………………………………
	□	…………………………………

**Additional questions for the diagnosis of acrophobia**		
9. Do you feel very intense fear or extremely strong fear when exposed to heights?	□	Yes
	□	No

10. I try in advance to avoid exposure to heights	□	No
	□	Yes

*Scoring instructions for the vHISS*.

*The scale is based on a set of eight questions for determining the severity of vHI. Two of the questions are lists: one of symptoms and one of triggers. Two additional questions are for the assessment of acrophobia*.

*1. Severity of vHI*.

*• Sum up the score of items 1–6*.

*• Add up the number of symptoms reported from List A (item 7). If there are less than 4 symptoms, add 0 to the total score; if there are 4 or more symptoms, add 1 to the total score*.

*• Likewise, add up the number of triggers from List B (item 8). If there are less than 4 triggers, add 0 to the total score. If there are 4–6 triggers, add 1 to the total score. For 7–9 triggers, add 2 to the total score. For 10 or more triggers, add 3 to the total score*.

*The sum of items 1–6, plus items of List A plus items of List B yields the total severity score*.

Severity score:

□ □ □ □ □ □ □ □ □ □ □ □ □

1 2 3 4 5 6 7 8 9 10 11 12 13

*2. Diagnosis of acrophobia*.

To meet DSM-V criteria for the diagnosis, one must have

*• At least one of the vegetative symptoms (a.–d.) from List A*.

*• Two other additional symptoms from List A*.

*• A positive response to item 6 (duration of at least 6 months) of the severity scale (yes)*.

*• A positive response to items 9 and 10 (yes)*.

Acrophobia:

**□ □**

yes no

## Materials and Methods

### Development of the Questionnaire by “Distilling” the Relevant Questions

The questionnaire was constructed from two more extensive questionnaires used in earlier representative epidemiological studies: one (*n* = 3,517) on the prevalence and determinants of vHI (5) and the other (*n* = 2,012) on clinical characteristics and psychiatric comorbidity patterns (4). Items were selected based on researchers’ clinical experience and based on a qualitative study in susceptible individuals (10).

We conducted a cross-sectional telephone survey of individuals representative of the general German population (*n* = 1,960). We considered this the best strategy for an optimal sample, although face-to-face interviews may provide more detailed individual information.

The questionnaire initially consisted of 16 questions (for original source, see Supplementary Material; note: a German translation was used since the survey was performed in Germany), the first of which was to determine the lifetime presence or absence of vHI or fear of heights: “Have you already experienced vHI (distressing instability when standing or moving) while looking from a height?” Only those who responded with “yes” continued. Eight questions were related to the severity of the condition (not strong, somewhat strong, moderately strong, quite strong, very strong), to how this susceptibility restricts the individual in daily activities and sports, and to the general impairment of quality of life. In addition to the specific triggers, bodily symptoms, frequency of occurrence, and the duration of the susceptibility (longer than 6 months?), behavioral consequences of the susceptibility were determined. Additionally, participants were asked if they had already undergone therapy or planned to do so. Based on practical considerations, all quantitative questions were scored on a 5-point scale: not at all, a little, moderately, quite a lot, and very much. We also asked participants to evaluate their overall susceptibility to heights (not strong, somewhat strong, moderately strong, quite strong, and very strong).

The survey was conducted according to the official directive on representative telephone-based market research involving the general population (infratest telephone master sample) and had a three-stage sampling design. It allowed us to select a limited sample of individuals aged 14 and older. This ensured an unbiased sample selection that excluded clustering effects. The multi-stratified, geographically based probability sampling of households allowed an additional random selection of defined targets. The study was performed by trained interviewers.

To create a scale to determine the severity of vHI, we chose 11 items (see Table 2) for testing. The objectivity of these 11 items was tested in a random subsample of 200 susceptible participants.

**Table 2:** **Items included in the partial credit model and their rescaling**.

		Score			
Item		0	1	2	3
Because of your visual height intolerance (vHI), how much difficulty did you recently have in sports (engaging in competitive and formal or informal organized games, alone or in a group)?	S1	Not at all	Moderately		
Because of your vHI, how much difficulty did you recently have in your daily activities?	S2	Not at all	Moderately	Quite a lot	
Because of your vHI, how much is your quality of life affected?	S3	Not at all	Moderately	Quite a lot	
I have already experienced once vHI when exposed to heights	S4	Occasionally	Often/always		
Now I have vHI that is …	S5	Less strong than before	Just as strong as before	Stronger than before	
I have/had vHI for longer than 6 months	S6	No	Yes		
Number of symptoms (List A)	S7	(<4)	(≥4)		
Number of different triggers (List B)	S8	(<4)	(4–6)	(7–9)	(10+)
Do you feel a very strong fear or extremely strong fear when exposed to vHI?	S9				
I try in advance to avoid exposure to heights	S10				
I quit as fast as possible all situations of acute exposure to heights	S11				

*Items S9–S11 did not show good fit to the model and were therefore eliminated*.

*Sums yield a raw score from 0 to 13 where 0 = least severely affected and 13 = most severely affected*.

To establish the psychiatric diagnosis of acrophobia, the following items had to be fulfilled [adapted from Ref. (4)]: intense fear; one from the items trembling, palpitation, inner agitation, sweating; two from the items light-headedness, to-and-fro vertigo, weakness in the knees, instability of stance and gait, queasy-stomach feeling, fearfulness, others; eliciting situations with visual height stimuli had to be actively avoided or endured with intense fear; a time criterion of at least 6 months of duration; any avoidance, anxious anticipitation, or distress in the feared situation had to interfere negatively with a person’s daily routine or social activities.

### Statistical Analyses

#### Descriptive Analysis

Percentages were used for categorical variables, while mean values and SDs were applied for numeric variables.

#### Rasch Analysis for Testing Objectivity

The Rasch model is a mathematical model that makes assumptions about response patterns resulting from rating scales. If data from a scale fits the expectations of a Rasch model, the criteria of specific objectivity are considered fulfilled. To fulfill the assumptions of a Rasch model, monotonicity, unidimensionality, and local independence of a scale have to be tested.

#### The Partial Credit Model (PCM)

It was used because it accounts for items with varying thresholds (11). The adequacy of the PCM was tested by a Likelihood ratio test.

#### Monotonicity

To investigate whether the various response levels of the scale work as anticipated, the empirical distribution of the thresholds was examined by looking at category probability curves. If thresholds are disordered, the difficulty of the response level does not correspond to the respective ability of the tested person. In the case of disordered thresholds, response levels were collapsed to resolve the problem.

#### Unidimensionality

Dimensionality was examined by residual principal components analysis (12). Items with positive factor loadings were considered candidates for a valid scale. The remaining items with negative factor loadings were examined by their ability to form a separate scale.

#### Local Independence

Local independence was tested by person-item residual correlation analysis. Items are locally independent, if the response to a specific item is not affected by the response to another item, once the latent underlying trait is accounted for (13). For example, the items “How much are you restricted in your activities of daily life” and “How much are you restricted in your sporting activities” are correlated. However, they are independent of each other, once the underlying trait, i.e., vHI, is accounted for (14).

#### Differential Item Functioning (DIF)

Each item of the scale has to be consistent and work independently of person factors, e.g., of sex or age of the respondent. Accordingly the DIF is tested across subgroups of person factors. For the analysis of DIF, the person factors age group, sex, and class intervals were treated as factors of an ANOVA. Their ANOVA statistics should be non-significant. If an item showed DIF, we removed it from the scale (15).

#### Fit of the Data to the Rasch Model

To assess overall fit, we investigated the item-trait interaction score. Good fit is indicated by a non-significant chi-square probability value (13). Additionally, two item-person interaction statistics (for item and for person fit) were used as indicators for overall fit. For both, a mean of zero and an SD of 1 indicate perfect fit to the model. On an individual level, the fit was investigated for person and item. The individual-person fit indicates how a person fits the Rasch model, i.e., individual-person fit residuals should be between −2.5 and +2.5. The individual-item-fit indicates how well an item fits the Rasch model. A non-significant individual-item-fit (based on a chi-squared test) indicates sufficient fit. Fit residuals such as the total of items deviating from the model were also assessed. They should lie within the range of ±2.5.

#### Internal Consistency

The person separation index (PSI) indicates how well the scale differentiates between patients. The PSI is close to 0, if all the persons are in a similar location; it approaches 1, the more the persons are spread across the item continuum (16). A PSI of >0.7 is generally found to be acceptable (17).

#### Targeting of the Scale

Targeting of items and persons was assessed using the Person-Item Threshold Distribution map, in which person locations are plotted together with item threshold locations. The significance level was set at 0.05. The Bonferroni method was applied as adjustment for multiple testing; it yielded a significance level of 0.05/*k*, where *k* is the number of tests carried out simultaneously.

#### Convergent Construct Validity

To assess convergent construct validity of the analyzed scale, the Spearman correlation coefficient of the sum score of the scale was calculated using the general question about how severely an individual was affected as external reference (Self-rated severity: “How severe is your vHI?”).

For this study, the questionnaire was used and analyzed in its German version (see Supplementary Material). We provide a preliminary English translation. So far, the English version has not been validated cross-culturally.

Descriptive analyses were carried out with SAS V9.2. RUMM 2030 was used for the Rasch analysis (Perth, RUMM Laboratory).

## Results

A total of 640 (females: 388) of the 1,960 (females: 1,073) participants indicated that they had experienced vHI; this corresponds to a prevalence of 32.7% (females: 36.1%; males: 28.4%). The overall evaluation comprised the data for all 640 susceptibles. The general state of health of the participants corresponded to that of the general population (see question 11). In the self-evaluation of the overall susceptibility, a relevant severity of vHI was estimated by 72% (*n* = 458) as being moderately strong (38%), quite strong (23%), or very strong (10%). The remaining 28% (*n* = 182) rated the severity as only somewhat strong or not strong. Sporting activities were felt restricted (moderately to very much) in 17%, everyday life activities in 14%; the quality of life was reduced in 12% (moderately to very much) and in an additional 22% by a little. The most frequent triggers were tower, scaffolding, roof, and ladder in about 70% of susceptibles, whereas everyday situations like standing on a balcony, a staircase, or looking out of a window elicited vHI in only 25–40% of susceptibles.

In most of the susceptibles vHI occurred occasionally (63%), in the others frequently or always. In about 60%, the severity of vHI varied over time becoming stronger than before in 38%. The most frequent bodily symptoms were malaise/queasy feeling in the stomach region (79%) and fearfulness (75%; 44% scaled their fearfulness as very or extremely strong), followed by instability of stance and gait (67%), inner agitation (61%), oppression (60%), and a mental image of falling (56%). The questions as to how susceptible individuals cope with vHI revealed that 70–80% try to avoid or quit as fast as possible situations that elicit distressing vHI or fear of heights. On the other hand, 24% reported that at least occasionally they expose themselves intentionally to heights. Acrophobia as defined in ICD-10 (6) and DSM-V (7) was fulfilled in 12.0% of susceptibles (*n* = 77; females: 49).

### Rasch Analysis

The objectivity of the items was investigated in a random subsample of 200 participants. Five of the initial items of the questionnaire showed disordered thresholds and were therefore rescored. Residual principal component analysis indicated unidimensionality. Three items showed local dependency with residual correlations above 0.3 (S9–S11); two items also showed DIF by age group (S10, S11). One item showed individual-item misfit (S9). Therefore, all three items were removed. The remaining eight items showed a good fit to the model. Iterative results are shown in Table 3.

**Table 3:** **Summary measures of model fit from the start to the final set**.

		Items				Persons				Item-trait-interaction		
		Location		Fit residual		Location		Fit residual				
						
	*N* (no. of items)	Mean	SD	Mean	SD	Mean	SD	Mean	SD	Chi-squared	*p*-Value	Person separation index*
Start	200 (11)	0.00	0.96	0.17	3.22	−0.15	0.80	−0.23	0.75	258.41 (99)	0.000	0.64
Mid-point	200 (10)	0.00	1.12	0.03	1.15	−0.16	1.15	−0.20	0.82	113.35 (90)	0.047	0.67
Final	200 (8)	0.00	0.90	0.11	1.09	−0.50	1.10	−0.16	0.82	72.25 (64)	0.224	0.61

*Summary statistics of a selected mid-point is provided to demonstrate the iterative approach*.

*Person separation indices can only be interpreted if there is sufficient overall fit of the data to the Rasch model, i.e., the p-value is non-significant.

**Figure 1:**
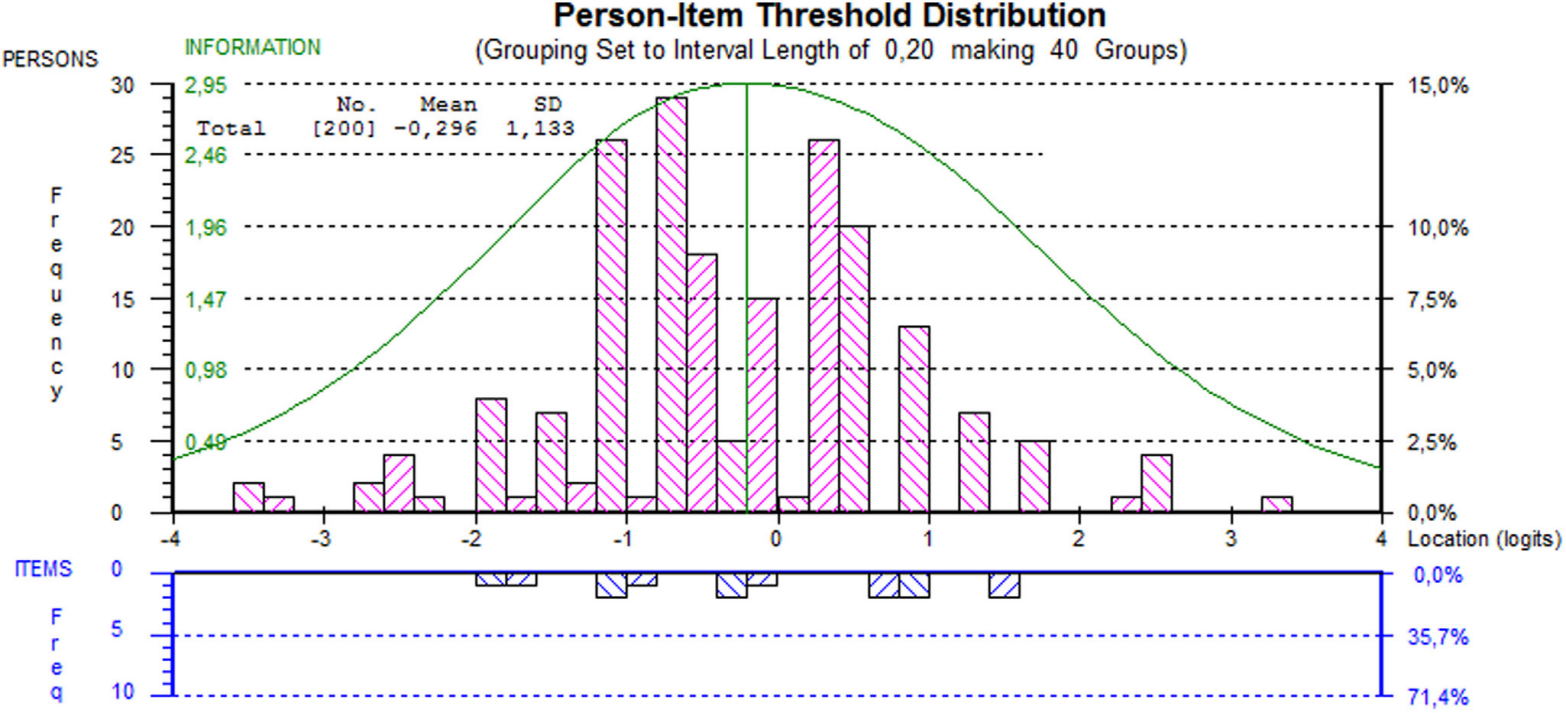
**Person-item threshold distribution map of the scale**. This indicates how persons and their abilities relate to item difficulty. The smaller the item difficulty, the higher the probability that an item will be completed. The information curve indicates the proportion of information provided by the items.

Person fit residuals indicated good individual fit. Internal consistency as quantified by the PSI was 0.61. Figure 1 shows the Person-Item Threshold Distribution map. This plot shows that the items are well distributed over the range of person abilities.

**Figure 2:**
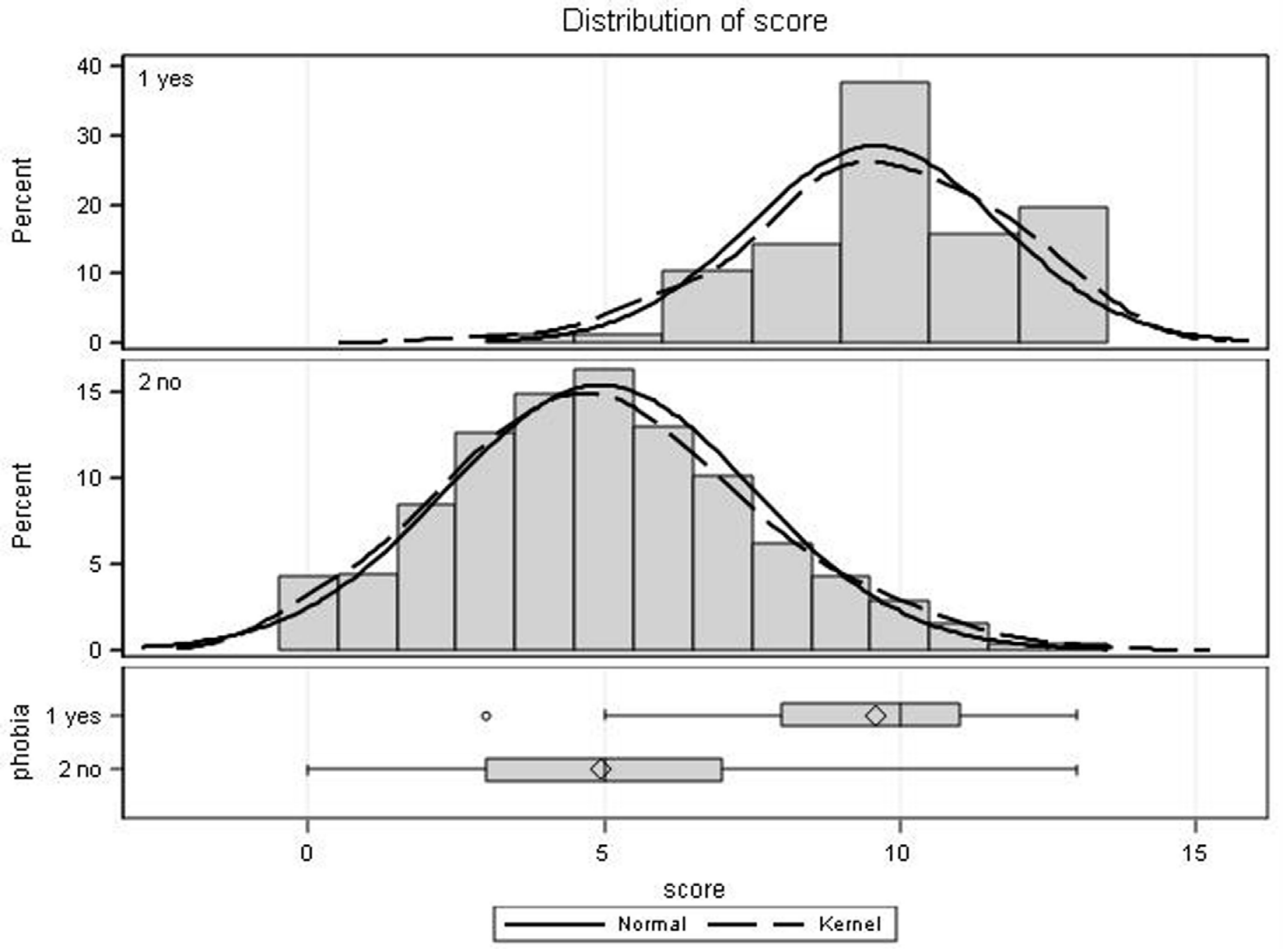
**Score distributions of the visual height intolerance severity scale stratified by presence (yes, *n* = 77) or absence (no, *n* = 562) of acrophobia**. Histograms are shown with overlaid normal and kernel densities. Diamonds in box plots indicate mean scores, whiskers display the maximum and minimum observations below and above the 1.5 interquartile range (upper fence), circles are maximum values above the upper fence.

The final scale yielded a raw score ranging from 0 to 13, where 0 = least severely affected and 13 = most severely affected. Convergent construct validity was calculated with the full sample of *n* = 640. The correlation of the raw score to self-rated severity was moderate (*r* = 0.46). Individuals with acrophobia scored significantly higher than those without acrophobia (mean score = 10 vs. mean score = 5, *p* < 0.0001) (see Figure 2).

## Discussion

**Figure 3:**
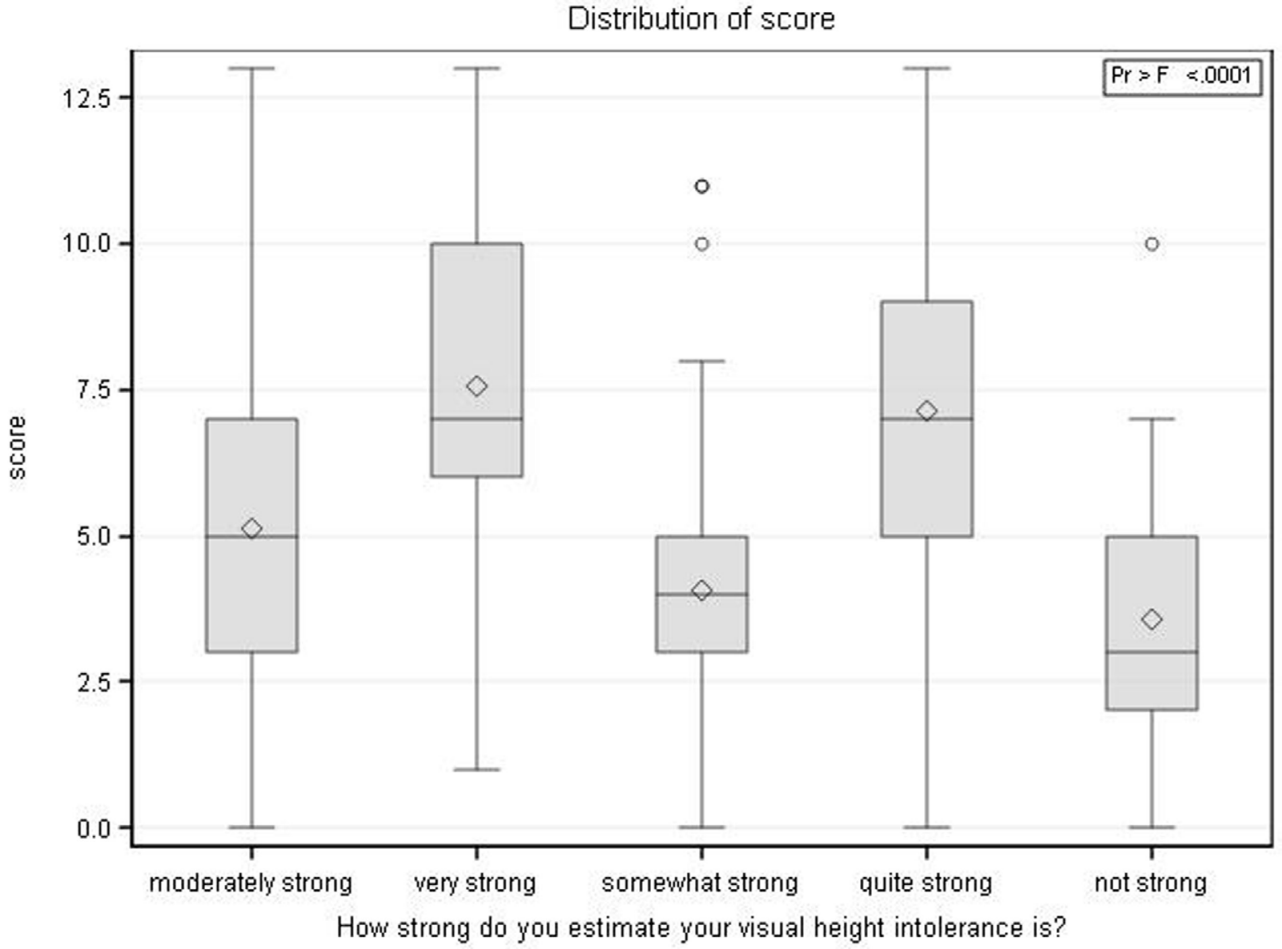
**Association of self-reported severity of visual height intolerance (vHI) and the newly developed vHI severity score**. Diamonds indicate mean scores, whiskers display the maximum and minimum observations below and above the 1.5 interquartile range (upper fence), circles are maximum values above the upper fence. The figure shows how subjects cannot reliably differentiate between “very strong” and “quite strong” or between “not strong” and “somewhat strong.”

The questionnaire (Table 1) revealed a lifetime prevalence of vHI of 32.7% with a gender preponderance of females (females: 36.1%; males: 28.4%). This is slightly higher than the lifetime prevalence determined in two earlier cross-sectional epidemiological studies, where it amounted to 28% (4, 5). The data include both the psychiatrically defined specific phobia fear of heights/acrophobia and the less severe forms of height intolerance. The frequency of acrophobia amounted to 12% of the 640 susceptible individuals, i.e., 3.9% of the total of 1,960 surveyed individuals. This corresponds to the frequency reported in earlier surveys of the general population which ranged from 3.1 to 6.4% (2–4). The validity of our questionnaire is further supported by the results that simply measure the self-reported general susceptibility to vHI and acrophobia (Figure 2). However, the metric interval scale of 0–13 points in our questionnaire calculated on the basis of eight questions better discriminates the severity of vHI in the individual, especially between less or more severely affected (Figure 2). As evident in Figure 3, a comprehensive self-report measure only correlates moderately with the raw score. Apparently, individuals cannot reliably discriminate between “very strong” and “quite strong” or between “not strong” and “somewhat strong.”

This is in line with the literature showing that adjectival scales are prone to bias because the meaning of terms such as “very,” “quite,” or “often” is influenced by individual interpretation and not always consistent (18).

One major advantage of the resulting interval scale is that a psychometrically sound sum score can be calculated that lends itself to parametric statistics.

To establish the diagnosis of acrophobia, questions 9 and 10 have to be added to the basic eight-question questionnaire. These questions are concerned with the symptoms of intense fear and an overt-behavioral avoidance when exposed to certain height stimuli. The distribution of the scores on the metric scale of the questionnaires of those individuals with acrophobia is separate and distinct from that of susceptibles without acrophobia (Figure 2), although there is some overlap. The latter demonstrates that the diagnostic criteria of acrophobia are not only fulfilled by those individuals with the highest scores. For this reason, we hesitate to categorize the metric scale from 0 to 13 into groups such as mild, medium, and severe.

The questionnaire has not been tested for its robustness in short-term repeated use or for its longitudinal validity, which is particularly important in follow-up and treatment studies. In an earlier study, the course of illness and the degree of social impairment were evaluated for a sample of 574 individuals with vHI, 128 of whom fulfilled the DSM-5 diagnostic criteria of acrophobia (19). More than two-thirds exhibited a persistent or worse course, particularly those with acrophobia. Female sex and psychiatric comorbidity also predicted an unfavorable course. This means that untreated vHI in adults is mostly a chronic, more or less distressing disability. This does not hold for prepubertal primary school children aged 8–10 years who also showed a prevalence of vHI of about one-third (20). However, in contrast to the adult-onset type of the condition, vHI in children appears to take a benign spontaneous course.

Our current experience with the above-described short questionnaire compared to the more comprehensive ones used in our previous epidemiological studies affirms its suitability as a tool for the potential applications listed in the Introduction.

## Ethics Statement

The study is based on an anonymous questionnaire with a verbal consent. Ethical approval was not required for this study in accordance with the institutional and national requirements. The study was performed in accordance with the ethical standards laid down in the 1964 Declaration of Helsinki.

## Author Contributions

DH and TB conceived and designed the study, interpreted the data, and wrote the manuscript. EG analyzed and interpreted the data, and wrote the manuscript.

## Conflict of Interest Statement

The authors declare that there are no conflicts of interest; there exist no financial or other relationships that have influenced the work. The reviewer, FC, and handling editor declared their shared affiliation, and the handling editor states that the process nevertheless met the standards of a fair and objective review.
