# Supplementary material for: A New Questionnaire for Estimating the Severity of Visual Height Intolerance and Acrophobia by a Metric Interval Scale
Source: Front Neurol. 2017 Jun 1;8:211. doi: 10.3389/fneur.2017.00211 (PMC5451500; doi:10.3389/fneur.2017.00211)
Supplement: Supplementary file 1 [file Presentation_1.PDF]

Initial questionnaire on visual height intolerance with 16 questions

Questionnaire on visual height intolerance

Name:____________________

Age:____

Sex: f 2☐ **m** 1☐

Question	Variable		
**1. Have you already experienced visual height intolerance** (distressing instability when standing or moving) **while looking from a height?**	V1	**Yes** 1☐	**No** 2☐
**Continue to fill out the rest of the questionnaire only if you answered “yes“.**		**Continue**	**Finished**
**2. How strong do you estimate your visual height intolerance is?**	V2	1☐<br>2☐<br>3☐<br>4☐<br>5☐	Not strong<br>Somewhat strong<br>Moderately strong<br>Quite strong<br>Very strong
**3. How much do you feel it interferes with sports?**	V3	1☐<br>2☐<br>3☐<br>4☐<br>5☐	Not at all<br>A little<br>Moderately<br>Quite a lot<br>Very much
**4. How much do you feel that visual height intolerance limits your activities in general, i.e. in everyday life?**	V4	1☐<br>2☐<br>3☐<br>4☐<br>5☐	Not at all<br>A little<br>Moderately<br>Quite a lot<br>Very much

Initial questionnaire on visual height intolerance with 16 questions

**5. How much do you feel it reduces your quality of life?**	V5	1☐<br>2☐<br>3☐<br>4☐<br>5☐	**Not at all**<br>**A little**<br>**Moderately**<br>**Quite a lot**<br>**Very much**

			Not at all	A little	Mode-rately	Quite a lot	Very much
**6. Visual height intolerance is induced by my…**<br><br>*(First 12 Codes Randomised)*	V6_1	**Standing on or climbing up a tower**	1☐	2☐	3☐	4☐	5☐
	V6_2	**Standing on or walking over a bridge**	1☐	2☐	3☐	4☐	5☐
	V6_3	**Standing on or walking up steps**	1☐	2☐	3☐	4☐	5☐
	V6_4	**Standing on or climbing up a ladder**	1☐	2☐	3☐	4☐	5☐
	V6_5	**Standing on or walking on a balcony**	1☐	2☐	3☐	4☐	5☐
	V6_6	**Looking out of a window**	1☐	2☐	3☐	4☐	5☐
	V6_7	**Standing or walking on a scaffolding**	1☐	2☐	3☐	4☐	5☐
	V6_8	**Standing or walking on a roof**	1☐	2☐	3☐	4☐	5☐
	V6_9	**Riding on a carousel or a Ferris wheel**	1☐	2☐	3☐	4☐	5☐
	V6_10	**Riding in a ski lift or gondola**	1☐	2☐	3☐	4☐	5☐
	V6_11	**Wandering/ mountain clim-bing**	1☐	2☐	3☐	4☐	5☐
	V6_12	**Rock climbing**	1☐	2☐	3☐	4☐	5☐

Initial questionnaire on visual height intolerance with 16 questions

	V6_13	**Other situations**	**1☐ 2☐ 3☐ 4☐ 5☐**
	V6_13Ao	**Examples**	________________________
**7. I have visual height intolerance when exposed to heights**	pV7	1☐<br>2☐<br>3☐	**... occasionally**<br>**... often/frequently**<br>**... always**
**8. Now I have visual height intolerance that is...**	V8	1☐<br>2☐<br>3☐	**... less strong than before**<br>**... just as strong as before**<br>**... stronger than before**
**9 I have/had visual height intolerance for longer than 6 months.**	V9	1☐<br>2☐	**No**<br>**Yes**
**10A. What <u>bodily</u> symptoms do you feel when exposed to heights? (Multiple answers possible)**<br><br>*(1 -5 Random)*<br><br>*0 = nicht genannt*<br>*1 = genannt*	pV101_1	☐	**Trembling**
	pV101_2	☐	**Palpitations**
	pV101_3	☐	**Inner agitation**
	pV101_4	☐	**Sweating**
	pV101_5	☐	**Moist hands**
	pV101_7	☐	**None of the above**
	pV101_9	☐	**Don't know, no response**
**10B. Do you feel very strong fear when exposed to heights?**	pV102	1☐<br>2☐	**Yes**<br>**No**
**10C. Do you feel any other symptoms during visual height intolerance?**	pV103	1☐<br>2☐	**Yes**<br>**No**
Filter: (only if answered "yes" to 10C)<br><br>**10D. What additional symptoms do you feel when experiencing visual height intolerance? (Multiple answers possible)**<br><br>*(First 10 Codes Randomised)*<br><br>*0 = not named*<br>*1 = named*	pV104_1	☐	**Giddiness**
	pV104_2	☐	**Postural (to-and-fro) dizziness**
	pV104_3	☐	**Weakness in the knees**
	pV104_4	☐	**Instability of stance and gait**
	pV104_5	☐	**Malaise/queasy feeling in the stomach region**
	pV104_6	☐	**Oppression**

Initial questionnaire on visual height intolerance with 16 questions

	pV104_7	☐	**Fearfulness**
	pV104_8	☐	**Mental image of falling**
	pV104_9	☐	**Gait disorder**
	pV104_10	☐	**Thinking blocked**
	pV104_11	☐	**Other, for example**
	pV104_96	☐	**None of the above**
	pV104_99	☐	**Don’t know, no response**
The following questions have to do with your general state of health. Your answers will allow us to understand how you feel and how you cope in your daily routine.			
**11. How would you describe your general state of health?**	V11	1☐<br>2☐<br>3☐<br>4☐<br>5☐	**Excellent**<br>**Very good**<br>**Good**<br>**Not so good**<br>**Bad**
The following questions are about how you cope with visual height intolerance.			
**12. I try in advance to avoid exposure to heights.**	V12	1☐<br>2☐	**No**<br>**Yes**
**13. I quit as fast as possible all situations of acute exposure to heights.**	V13	1☐<br>2☐	**No**<br>**Yes**
**14. I intentionally expose myself to heights.**	V14	1☐<br>2☐<br>3☐	**No**<br>**Occasionally**<br>**Yes**
**15. Have you already finished a training program to cope with visual height intolerance?**<br><br>*Filter: Only when 15 was No*	V15	1☐<br>2☐	**Yes**<br>**No**
**16. Are you planning on training to cope with visual height intolerance?**	pV16	1☐<br>2☐	**Yes**<br>**No**
