# Supplementary material for: A New Questionnaire for Estimating the Severity of Visual Height Intolerance and Acrophobia by a Metric Interval Scale
Source: Front Neurol. 2017 Jun 1;8:211. doi: 10.3389/fneur.2017.00211 (PMC5451500; doi:10.3389/fneur.2017.00211)
Supplement: Supplementary file 2 [file Presentation_2.PDF]

A new questionnaire for estimating the severity of visual height intolerance and acrophobia by a metric interval scale

Huppert D, Grill E, Brandt T; Frontiers in Neurology 2017; DOI: 10.3389/fneur.2017.00211

Visual Height Intolerance Severity Scale (vHISS)		
**Question**		
**Have you already experienced visual height intolerance while looking from a height? (distressing instability when standing or moving)**	**Yes** ☐ ↓	**No** ☐ ↓
**Continue to fill out the rest of the questionnaire only if you answered "yes".**	**Continue**	**Finished**
**1. Because of your visual height intolerance, how much difficulty did you recently have doing sports?**	0☐<br>1☐	**No difficulty**<br>**Any difficulty (a little/moderately/quite a lot/very much)**
**2. Because of your visual height intolerance, how much difficulty did you recently have in your daily activities?**	0☐<br>1☐<br>2☐	**No difficulty**<br>**A little**<br>**Moderately/quite a lot/very much**
**3. Because of your visual height intolerance, how much is your quality of life affected?**	0☐<br>1☐<br>2☐	**Not at all**<br>**A little**<br>**Moderately/quite a lot/very much**
**4. I have visual height intolerance when exposed to heights.**	0☐<br>1☐	**… occasionally**<br>**… often/frequently/always**
**5. Now I have visual height intolerance that is…**	0☐<br>1☐<br>2☐	**… less strong than before**<br>**… just as strong as before**<br>**… stronger than before**
**6. I have/had visual height intolerance for longer than 6 months.**	0☐<br>1☐	**No**<br>**Yes**

List A

**7. What bodily symptoms do you feel when exposed to heights?** (Multiple answers possible)	☐	**a. Trembling**
	☐	**b. Palpitations**
	☐	**c. Inner agitation**
	☐	**d. Sweating/ Moist hands**
	☐	**e. Lightheadedness**
	☐	**f. Postural (to-and-fro) dizziness**
	☐	**g. Weakness in the knees**
	☐	**h. Instability of stance and gait**
	☐	**i. Malaise/queasy feeling in the stomach**
	☐	**k. Oppression**
	☐	**l. Fearfulness**
	☐	**m. Mental image of falling**
	☐	**m. Gait disorder**
	☐	**o. Others…………………………..**
	☐	**None of the above**

List B

**8. Visual height intolerance is induced by my…** (Multiple answers possible)	☐	**Standing on or climbing up a tower**
	☐	**Standing on or walking over a bridge**
	☐	**Standing on or walking up steps**
	☐	**Standing on or climbing up a ladder**
	☐	**Standing on or walking on a balcony**
	☐	**Looking out of a window**
	☐	**Standing or walking on a scaffolding**
	☐	**Standing or walking on a roof**
	☐	**Riding on a carousel or a Ferris wheel**
	☐	**Riding in a ski lift or gondola**
	☐	**Hiking/mountain climbing**
	☐	**Rock climbing**
	☐	**Other situations. If yes, please name:**
	☐	**……………………………………….**
	☐	**……………………………………….**

Additional questions for the diagnosis of acrophobia

**9. Do you feel very intense fear or extremely strong fear when exposed to heights?**	☐<br>☐	**Yes**<br>**No**
**10. I try in advance to avoid exposure to heights.**	☐<br>☐	**No**<br>**Yes**

Scoring instructions for the visual Height Intolerance Severity Scale
The scale is based on a set of eight questions for determining the severity of visual height intolerance. Two of the questions are lists: one of symptoms and one of triggers. Two additional questions are for the assessment of acrophobia.

1. Severity of Visual Height Intolerance
- Sum up the score of items 1 to 6.
- Add up the number of symptoms reported from List A (item 7). If there are less than 4 symptoms, add 0 to the total score; if there are 4 or more symptoms, add 1 to the total score.
- Likewise, add up the number of triggers from List B (item 8). If there are less than 4 triggers, add 0 to the total score. If there are 4 to 6 triggers, add 1 to the total score. For 7 to 9 triggers, add 2 to the total score. For 10 or more triggers, add 3 to the total score.

The sum of items 1 to 6, plus items of List A plus items of List B yields the total severity score.

Severity score

☐	☐	☐	☐	☐	☐	☐	☐	☐	☐	☐	☐	☐
**1**	**2**	**3**	**4**	**5**	**6**	**7**	**8**	**9**	**10**	**11**	**12**	**13**

2. Diagnosis of Acrophobia
To meet DSM-V criteria for the diagnosis, one must have:
- at least one of the vegetative symptoms (a. – d.) from List A
- two other additional symptoms from List A
- a positive response to item 6 (duration of at least 6 months) of the severity scale (yes)
- a positive response to items 9 and 10 (yes)

Acrophobia

☐	☐
**yes**	**no**

A new questionnaire for estimating the severity of visual height intolerance and acrophobia by a metric interval scale

Huppert D, Grill E, Brandt T; Frontiers in Neurology 2017; DOI: 10.3389/fneur.2017.00211

Fragebogen zur Schwere von visueller Höhenintoleranz (vHISS)

Frage		
**Haben Sie bereits Höhenschwindel beim Blick aus der Höhe erlebt?** (Stand- und/oder Bewegungsunsicherheit)	Ja ☐	Nein ☐
Nur bei "ja" bitte Fragebogen weiter ausfüllen.	↓ Fortfahren	⊥ Beenden
1. Inwieweit sehen Sie sich durch Ihren Höhenschwindel in Ihren sportlichen Aktivitäten beeinträchtigt?	0☐<br>1☐	Gar nicht<br>Ein wenig, mäßig, ziemlich, sehr
2. Inwieweit sehen Sie sich durch Ihren Höhenschwindel in Ihren allgemeinen Aktivitäten, d.h. im täglichen Leben, beeinträchtigt?	0☐<br>1☐<br>2☐	Gar nicht<br>Ein wenig<br>Mäßig, ziemlich, sehr
3. Inwieweit sehen Sie sich durch Ihren Höhenschwindel in Ihrer Lebensqualität beeinträchtigt?	0☐<br>1☐<br>2☐	Gar nicht<br>Ein wenig<br>Mäßig, ziemlich, sehr
4. Höhenschwindel tritt bei mir bei Höhenreizen auf	0☐<br>1☐	… gelegentlich<br>… oft/häufig/immer
5. Höhenschwindel ist jetzt bei mir …	0☐<br>1☐<br>2☐	… weniger stark als früher<br>… genauso stark wie früher<br>… stärker als früher
6. Höhenschwindel habe/hatte ich länger als 6 Monate.	0☐<br>1☐	Nein<br>Ja

A new questionnaire for estimating the severity of visual height intolerance and acrophobia by a metric interval scale
Huppert D, Grill E, Brandt T; Frontiers in Neurology 2017; DOI: 10.3389/fneur.2017.00211

Liste A

**7. Welche körperlichen Symptome verspüren Sie bei Höhenreizen?** (Mehrfachantworten möglich)	☐	**Zittern**
	☐	**Herzrasen**
	☐	**Innere Unruhe**
	☐	**Schwitzen / Feuchte Hände**
	☐	**Benommenheit**
	☐	**Schwankschwindel**
	☐	**Schwäche in den Knien**
	☐	**Stand-/Trittunsicherheit**
	☐	**Unwohlsein/Flaues Bauchgefühl**
	☐	**Beklemmungsgefühl**
	☐	**Ängstlichkeit**
	☐	**Sturzvorstellung**
	☐	**Gangstörung**
	☐	**Andere………………………….**
	☐	**Keine davon**

Liste B

**8. Höhenschwindel wird bei mir ausgelöst durch ...** (Mehrfachantworten möglich)	☐	**Stehen oder Gehen auf einem Turm**
	☐	**Stehen oder Gehen auf einer Brücke**
	☐	**Stehen oder Gehen auf einer Treppe**
	☐	**Stehen oder Gehen auf einer Leiter**
	☐	**Stehen oder Gehen auf einem Balkon**
	☐	**Blick aus Fenster**
	☐	**Stehen oder Gehen auf einem Gerüst**
	☐	**Stehen oder Gehen auf einem Dach**
	☐	**Karussel/ Riesenrad fahren**
	☐	**Lift/Gondel fahren**
	☐	**Wandern/ Bergsteigen**
	☐	**Klettern**
	☐	**Sonstige Situationen**
	☐	**und zwar:**
	☐	**…………………………………………..**
		**…………………………………………..**

Zusatzfragen für die Diagnose Akrophobie

**9. Verspüren Sie bei Höhenreizen sehr starke Angst?**	☐<br>☐	**Ja**<br>**Nein**
**10. Ich versuche im Vorfeld, Höhenreize zu umgehen.**	☐<br>☐	**Nein**<br>**Ja**

Instruktionen zur Auswertung der Schweregradskala

Die Skala basiert auf acht Fragen zur Ermittluung der Schwere einer visuellen Höhenintoleranz. Zwei der Fragen sind Listen: eine der Symptome und eine der Auslösesituationen. Zwei zusätzliche Fragen dienen zur Diagnose einer Akrophobie.

1. Schwere der visuellen Höhenintoleranz

- Zählen Sie die Punkte der Fragen 1 – 6 zusammen.
- Zählen Sie die Anzahl der Symptome aus Liste A zusammen (Frage 7). Bei weniger als vier Symptomen addieren Sie 0 zu der Skala, bei mehr als vier Symptomen addieren Sie 1 zu der Skala.
- Zählen Sie die Anzahl der Auslösesituationen aus Liste B zusammen (Frage 8). Bei weniger als vier Auslösern addieren Sie 0 zu der Skala, bei mehr als vier Auslösern 1. Bei 7 bis 9 Auslösesituationen addieren Sie 2, bei mehr als 10 Auslösern 3 zu der Gesamtskala.

Die Summe der Fragen 1 - 6, plus die Punkte aus Liste A plus die Punkte aus Liste B ergibt den Gesamtwert der Skala

Schweregrad (Skala)

☐	☐	☐	☐	☐	☐	☐	☐	☐	☐	☐	☐	☐
**1**	**2**	**3**	**4**	**5**	**6**	**7**	**8**	**9**	**10**	**11**	**12**	**13**

2. Diagnose einer Akrophobie

Für die Diagnosekriterien von ICD-10 bzw. DSM-5 müssen vorliegen:

- Mindestens ein vegetatives Symptom (a. – d.) der Liste A
- Zwei weitere Symptome der Liste A
- Eine positive Antwort auf Frage 6 (Dauer mindestens 6 Monate) der Schweregradskala (“ja”)
- Eine positive Antwort auf die Fragen 9 und 10 (“ja”)

Akrophobie

☐	☐
**ja**	**nein**
